# Acupressure at the Meridian Acupoint Xiyangguan (GB33) Influences Near-Infrared Spectroscopic Parameters (Regional Oxygen Saturation) in Deeper Tissue of the Knee in Healthy Volunteers

**DOI:** 10.1155/2013/370341

**Published:** 2013-02-07

**Authors:** Gerhard Litscher, Michael Ofner, Wei He, Lu Wang, Ingrid Gaischek

**Affiliations:** ^1^Stronach Research Unit for Complementary and Integrative Laser Medicine, Research Unit of Biomedical Engineering in Anesthesia and Intensive Care Medicine and TCM Research Center Graz, Medical University of Graz, Auenbruggerplatz 29, 8036 Graz, Austria; ^2^Department of Meridians, Institute of Acupuncture and Moxibustion, China Academy of Chinese Medical Sciences, Beijing 100700, China; ^3^Department of Sports Physiology, University of Vienna, 1150 Vienna, Austria

## Abstract

Up to now, it is still unknown whether microcirculation of deeper peripheral tissue (knee) can be modulated by acupuncture or acupressure on a meridian acupoint. The goal of this pilot study was to investigate possible effects of acupressure at the Xiyangguan acupoint (GB33) on the regional oxygen saturation of the deeper knee tissues by near-infrared spectroscopy (NIRS). Twelve healthy volunteers with a mean age of 23.8 ± 1.6 years were investigated. Acupressure stimulation was performed for 5 minutes at the Xiyangguan acupoint. The results of the controlled study showed a significant increase of the values of regional oxygen saturation on the stimulated side of the knee (*P* = 0.033), whereas the opposite side on the same knee showed insignificant changes. These results may serve as a valuable basis for monitoring a possible therapeutic effect (e.g., after Khalifa therapy) in patients with knee problems.

## 1. Introduction 

As one of the complementary and alternative treatments (CAMs), acupuncture or acupressure has been utilized to improve health in Asia and Western countries. Although the mechanism of acupuncture remains still unknown in detail, acupuncture stimulation was confirmed to increase the blood flow velocity of the peripheral arterioles [1]. Up to now, studies have shown that the blood circulation of the body surface can be modulated by acupuncture or acupressure [2, 3]. But it is still unknown whether the microcirculation of the deeper peripheral tissue can be modulated by acupuncture or acupressure.

Near-infrared spectroscopy (NIRS) is a spectroscopic method that uses the near-infrared region of the electromagnetic spectrum (from about 800 nm to 2500 nm). Medical applications of NIRS center on the noninvasive measurement of the amount and oxygen content of hemoglobin [4]. The advantage of NIRS is that it can typically penetrate much deeper into a sample than mid-infrared radiation [5]. 

Acupressure treatment normally uses fingertips, rather than needles, to stimulate acupoints on the skin and has been shown to be a successful treatment for a variety of medical disorders. 

The goal of the present pilot study was to investigate possible effects of acupressure at the Xiyangguan acupoint (GB33) on the regional oxygen saturation of the knee tissues at a depth of 2–4 cm, by using NIRS. 

## 2. Materials and Methods

### 2.1. Healthy Volunteers

Twelve healthy volunteers (5 females, 7 males) with a mean age ± SD of 23.8 ± 1.6 years were investigated at the Medical University of Graz. None of the subjects had any neurological or cardiovascular disorders, and none of them was taking any medication. They were informed about the nature of the investigation and were paid for their participation. The study was approved by the local ethics committee, and all participants provided written informed consent.

### 2.2. Acupressure

Acupressure stimulation was performed at the Xiyangguan (GB33) acupoint. This point is located on the lateral side of the knee, 3 cun (*cun* is a relative body measure; 1 cun corresponds to the breadth of the distal phalanx of the thumb) directly proximal to Yanglingquan (GB34), at the depression superior to the lateral epicondyle of the femur, between the femur and the tendon of biceps femoris. Stimulation of Xiyangguan is indicated in cases of swelling and pain in the knee caused by inflammatory processes [6]. To assess the reliability and validity of acupressure, pressure was applied by the same Chinese medical doctor experienced in Traditional Chinese Medicine (TCM). The thumb pressure was steady and estimated to be about 3 × 10^5^ Pa (mean force measured ~ 30 N/cm^2^), as described in [7].

### 2.3. Evaluation Parameters

**Figure 1:**
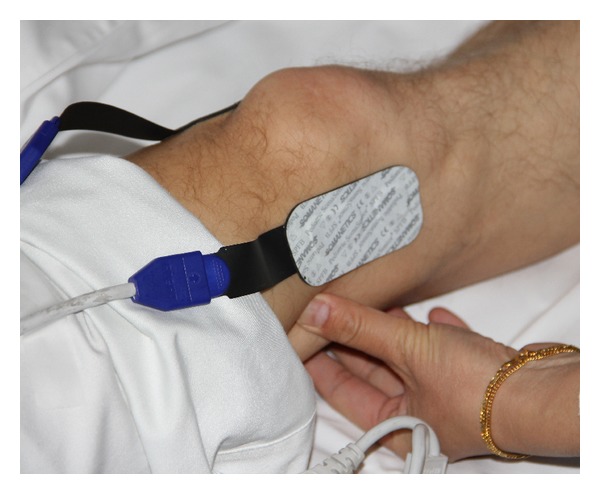
Measurement site with applied sensors during acupressure stimulation.

For the measurement of the regional oxygen saturation (rSO_2_), a two-channel INVOS 5100 Oximeter (Somanetics Corp., Troy, USA) was used. The principle of this system is based on NIRS technology, which is a noninvasive method for measuring regional oxygenation through the intact skin, which has been applied successfully in research and numerous clinical indications for many years [8]. Near-infrared light (730 and 805 nm) is emitted through the skin and after passing different kinds of tissue (muscle and bone) the returned light is detected at two distances from the light source (3 and 4 cm). Based upon this principle, the spectral absorption of blood in deeper structures (2–4 cm) can be determined and defined as rSO_2_. Before starting the measurement, the skin was cleaned with the enclosed skin-prep pad. Then two sensors were applied below the right and left lateral sides of the patella of the right leg (see Figure 1). To minimize external light influence, the knee was covered with a black cloth during the recording and stimulation procedure. After a resting time of five minutes, the rSO_2_ data were recorded.

### 2.4. Procedure

**Figure 2:**
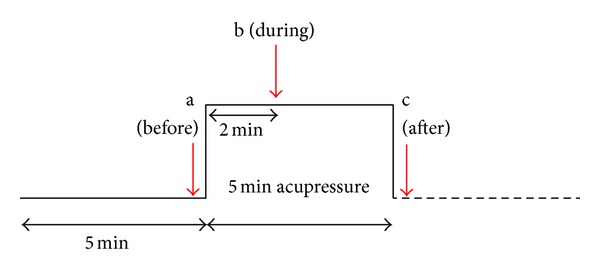
Measurement profile of the volunteer study. The measurement points are indicated (a–c).

The persons were investigated in a supine position. Acupressure started after a 5-minute resting phase. The measurement profile is shown in Figure 2. Three measurement points were evaluated (a, immediately before starting the stimulation; b, 2 min after starting the stimulation; and c, immediately after the end of the 5-minute stimulation period.

The study was performed as a controlled study. The parameter rSO_2_ was measured at two different locations at the stimulated knee. Location 1 (acupressure side) was in a distance of 2 cm from the acupuncture point Xiyangguan (comp. Figure 1). Location 2 (serving as control) was on the same knee, on the opposite side of the patella. We did not measure the results of other acupuncture points within this study.

### 2.5. Statistical Analysis

The rSO_2_ values of both the acupressure and the opposite side were tested with one way repeated measures ANOVA (SigmaPlot 12.0, Systat Software Inc., Chicago, USA). The Holm-Sidak method was used for post-hoc analysis. The level of significance was defined as *P* < 0.05.

## 3. Results

**Figure 3:**
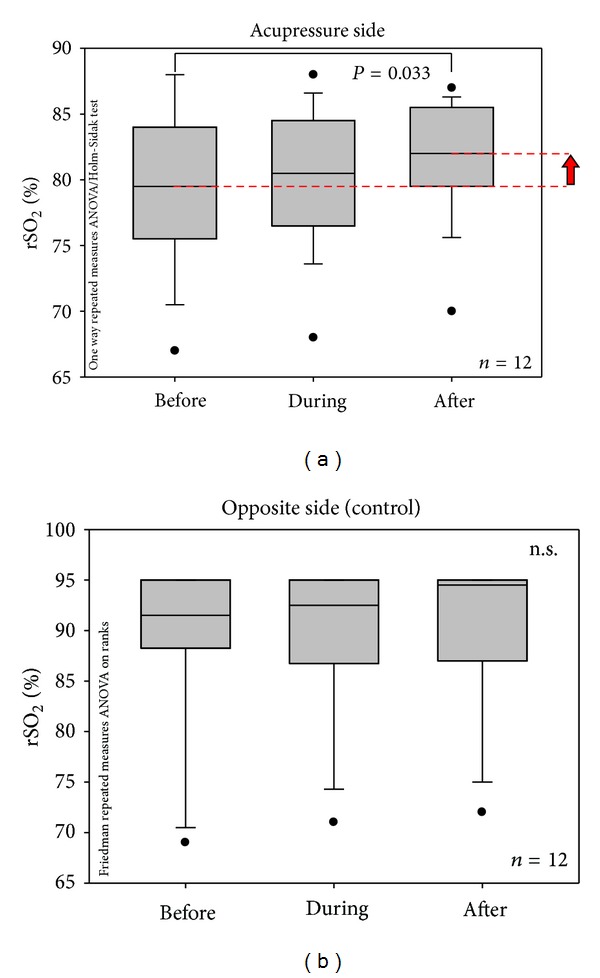
(a) Box plot presentation of changes of regional oxygen saturation values in 12 healthy volunteers before, during, and after acupressure (comp. Figure 2) on the stimulated side (acupressure side). Note the significant increase immediately after the 5-minute acupressure phase; the median of the rSO_2_ was increased by 2.5% compared to baseline values. The ends of the boxes define the 25th and 75th percentiles with a line at the median, and error bars defining the 10th and 90th percentiles. (b) Box plot presentation of changes of regional oxygen saturation values in 12 healthy volunteers before, during, and after acupressure (comp. Figure 2) on the opposite (control) side. For further explanations, see Figure 3(a).

All subjects completed the study, and the measurements could be performed without any technical problems. Stimulation was perceived as painless and not discomforting.  Figure 3(a) shows the increase of the rSO_2_ values during and after acupressure being applied to the acupoint Xiyangguan in all 12 healthy volunteers.

The values of rSO_2_ of the opposite side are presented in Figure 3(b). No statistically significant differences were found on this side, although the median was increased during and after acupressure.

## 4. Discussion

Acupressure is a noninvasive strategy used to manage multiple symptoms in a variety of patient populations [9], including relieving pain [10], managing nausea and vomiting [11], and many other applications.

The acupuncture point Xiyangguan (GB33) is located on the gall bladder meridian. This meridian runs along the lateral aspect of the thigh and knee, going further downward along the anterior border of the fibula. As mentioned in the methods section, GB33 is located lateral to the knee joint. It is a conventional acupoint in the treatment of knee osteoarthritis [12]. In the present study, the oxygen saturation of a healthy knee could be affected by acupressure at GB33 in a depth of 2–4 cm. Our results are in accordance with the meridian theory, which states that firstly, the area along the meridian will be affected by stimulating acupoints on the meridian, and secondly, the function of local tissues can be affected by stimulating nearby acupoints [13].

In recent years, we have investigated the relationship between acupuncture and cerebral microcirculation using NIRS. In three healthy male volunteers, acupuncture at specific acupuncture points led to an increase in oxygenated hemoglobin and in the tissue oxygenation index. However, needling and laser puncture at placebo points did not produce the same effect on cerebral oxygenation [14]. In 16 adult volunteers, a significant decrease was found in oxyhemoglobin after needle insertion and stimulation, accompanied by an increase in deoxyhemoglobin [15]. The results suggest that NIRS technology may be useful in visualizing and quantifying the cerebral vascular effects of acupuncture and acupuncture-like stimulation on microcirculation [16]. 

The primary application of NIRS to the human body uses the fact that the transmission and absorption of NIR light in human body tissues contains information about hemoglobin concentration changes. NIRS can be used to quantify blood flow, blood volume, oxygen consumption, reoxygenation rates, and muscle recovery time [17]. In comparison with laser Doppler flowmetry (LDF), NIRS can typically penetrate much deeper into a sample. NIRS can penetrate the tissue in a depth of 2–4 cm, whereas LDF can only penetrate the tissue in a depth of about 1–3 mm. NIRS is more sensitive than LDF with regard to detecting changes in tissue inflow [18]. Besides, NIRS systems are usually portable, even wireless instrumentation is available, which enables investigations in freely moving subjects [19]. It has also been suggested as a method for arthroscopic evaluation of low grade degenerated cartilage lesions [20].

To the best of our knowledge, this is the first study which evaluates the effects of acupressure on the regional blood oxygenation of the knee tissues using NIRS.

Studies have investigated the mechanism of acupuncture or acupressure with respect to peripheral microcirculation. The blood flow velocity was found to be increased after continuous digital acupressure. It has also been suggested that cyclic Guanosine Monophosphate (cGMP) mediates the signaling functions of nitric oxide (NO) to improve local microcirculation [21]. A neurovascular transmission model for the acupuncture-induced NO effect has been proposed by Hsiao and Tsai [22]. In this model, the acupuncture stimulus is able to influence connective tissue via mechanical force transfer to the extracellular matrix (ECM). Through the ECM, the mechanotransduction stimulus can be translated or travel from the acupuncture points including local tissue and cells. Cells in the local tissue that have received mechanotransduction induce different types of NO production to induce changes in blood flow and local circulation [22]. By assessing the responses of arteriolar blood flow to acupuncture stimulation in rabbits, it was found that the arteriolar diameter significantly increased to 131% ± 14% in the acupuncture group when compared with the pretreatment value. Blood flow velocity and blood flow rate showed similar trends. The treatment effect remained manifest for 40–50 min after the end of stimulation and irradiation [23]. 

Our preliminary study has some limitations. We did not measure environmental temperature which could possibly also affect the results, but this is not very probable. It is also possible that the articular cavity can influence the measurement results, and this could also be a reason for the variation among the subjects before the procedure. Moreover, the informative value of our results may be compromised by the fact that already baseline values differ strongly between the acupressure side and the opposite (control) side, being much higher on the opposite side.

Acupressure is a noninvasive therapy, which is readily accepted by people with needle phobia. It can be used similar to acupuncture, for example, in the treatment of knee osteoarthritis or other diseases related to the knee [24].

## 5. Conclusion

The following conclusion can be drawn from the present pilot study. The values of regional oxygen saturation (rSO_2_) on the stimulated side of the knee were significantly increased immediately after acupressure stimulation, whereas the opposite (control) side on the same knee showed insignificant changes. Further investigations with a four-channel NIRS system for measurements on both knees are in progress.
